# Digital Approaches to Remote Pediatric Health Care Delivery During the COVID-19 Pandemic: Existing Evidence and a Call for Further Research

**DOI:** 10.2196/20049

**Published:** 2020-06-25

**Authors:** Sherif M Badawy, Ana Radovic

**Affiliations:** 1 Department of Pediatrics Northwestern University Feinberg School of Medicine Chicago, IL United States; 2 Division of Hematology, Oncology and Stem Cell Transplant Ann & Robert H Lurie Children's Hospital of Chicago Chicago, IL United States; 3 Department of Pediatrics University of Pittsburgh Medical Center Children’s Hospital of Pittsburgh University of Pittsburgh School of Medicine Pittsburgh, PA United States

**Keywords:** coronavirus, COVID-19, SARS-CoV-2, pandemic, outbreak, public health, pediatric, children, adolescents, telehealth, telemedicine, digital, interventions, digital health, digital medicine, mobile health, mHealth, eHealth, health care delivery

## Abstract

The global spread of the coronavirus disease (COVID-19) outbreak poses a public health threat and has affected people worldwide in various unprecedented ways, both personally and professionally. There is no question that the current global COVID-19 crisis, now more than ever, is underscoring the importance of leveraging digital approaches to optimize pediatric health care delivery in the era of this pandemic. In this perspective piece, we highlight some of the available digital approaches that have been and can continue to be used to streamline remote pediatric patient care in the era of the COVID-19 pandemic, including but not limited to telemedicine. *JMIR Pediatrics and Parenting* is currently publishing a COVID-19 special theme issue in which investigators can share their interim and final research data related to digital approaches to remote pediatric health care delivery in different settings. The COVID-19 pandemic has rapidly transformed health care systems worldwide, with significant variations and innovations in adaptation. There has been rapid expansion of the leveraging and optimization of digital approaches to health care delivery, particularly integrated telemedicine and virtual health. Digital approaches have played and will play major roles as invaluable and reliable resources to overcome restrictions and challenges imposed during the COVID-19 pandemic and to increase access to effective, accessible, and consumer-friendly care for more patients and families. However, a number of challenges remain to be addressed, and further research is needed. Optimizing digital approaches to health care delivery and integrating them into the public health response will be an ongoing process during the current COVID-19 outbreak and during other possible future pandemics. Regulatory changes are essential to support the safe and wide adoption of these approaches. Involving all relevant stakeholders in addressing current and future challenges as well as logistical, technological, and financial barriers will be key for success. Future studies should consider evaluating the following research areas related to telemedicine and other digital approaches: cost-effectiveness and return on investment; impact on quality of care; balance in use and number of visits needed for the management of both acute illness and chronic health conditions; system readiness for further adoption in other settings, such as inpatient services, subspecialist consultations, and rural areas; ongoing user-centered evaluations, with feedback from patients, families, and health care providers; strategies to optimize health equity and address disparities in access to care related to race and ethnicity, socioeconomic status, immigration status, and rural communities; privacy and security concerns for protected health information with Health Insurance Portability and Accountability Act (HIPAA)–secured programs; confidentiality issues for some specific populations, especially adolescents and those in need of mental health services; early detection of exposure to violence and child neglect; and integration of training into undergraduate and graduate medical education and subspecialty fellowships. Addressing these research areas is essential to understanding the benefits, sustainability, safety, and optimization strategies of telemedicine and other digital approaches as key parts of modern health care delivery. These efforts will inform long-term adoption of these approaches with expanded dissemination and implementation efforts.

## The Burden of the COVID-19 Pandemic

The global spread of COVID-19, the disease caused by severe acute respiratory syndrome coronavirus 2 (SARS-CoV-2), has posed a public health threat and has affected people worldwide in various unprecedented ways, both personally and professionally. COVID-19 infection is asymptomatic in many cases; however, in more serious cases, it can lead to severe acute respiratory syndrome, which requires mechanical ventilation, circulatory support, and intensive care unit management. Overall, children have been much less affected than adults in terms of both prevalence and disease severity [1], although the long-term effects are as yet unknown and the evidence base is evolving. The World Health Organization declared COVID‐19 a pandemic on March 11, 2020 [2]. COVID-19 has been spreading rapidly worldwide among children and adults in 187 countries; according to the Johns Hopkins Coronavirus Resource Center, there have been 7,397,349 confirmed cases and 417,133 global deaths as of June 11, 2020 [3]. These numbers continue to increase every day, especially with the high reproductive number of SARS-CoV-2 [4]. Countries with the highest disease burden, particularly fatalities, include the United States, United Kingdom, Brazil, Italy, France, Spain, and Mexico [3].

Several strategies have been implemented worldwide to limit the spread of COVID-19 infection, such as social (ie, physical) distancing and local or national stay-at-home mandates. These strategies, although necessary, have led not only to disruption of people’s normal routines or daily life in different ways but also to significant financial challenges to our society and economy across almost all sectors, including health care. Just a few of the many disruptions that are more relevant to pediatrics are lower availability of child care sources due to the closure of day care centers, preschool centers, and schools; homeschooling of children and adolescents; parents or caregivers who are working remotely, have been furloughed, or have temporarily or permanently lost their jobs; limited ability to decrease individual density in outpatient settings, such as pediatricians’ private offices and hospital clinics; postponement of elective surgeries, procedures, and imaging studies; avoidance of emergency rooms by children and families who may be in need of health care; and missed appointments for routine children’s vaccinations. All these challenges have resulted in increased levels of stress and uncertainty in the lives of our patients and their families.

## Digital Approaches to Pediatric Health Care Delivery

There is no question that the current global COVID-19 crisis, now more than ever, is underscoring the importance of leveraging digital approaches to optimize pediatric health care delivery in the era of this pandemic. Given the restrictions and limitations of in-person or face-to-face visits, many patients, families, and clinicians, including pediatricians, are increasingly realizing the potential of these tools. Additionally, access to personal technology is increasingly ubiquitous [5-7]. Moreover, there is growing evidence to support the feasibility, acceptability, and efficacy of digital behavioral interventions in pediatric populations [8-16], although economic evaluations are lacking [17]. However, concerns remain related to inequity of access to the internet, especially broadband connections; therefore, promotion of digital and telehealth equity is urgently needed. In this perspective piece, we highlight some of the available digital approaches that have been and can continue to be used to streamline remote pediatric patient care in the era of the COVID-19 pandemic, including and beyond telemedicine alone. *JMIR Pediatrics and Parenting* is currently publishing a COVID-19 special theme issue for investigators to share their interim and final research data related to digital approaches to remote pediatric health care delivery in different settings, such as telemedicine or telehealth, web-based interventions, mobile apps, wearable devices, and other novel digital strategies [Multimedia Appendix 1] [18].

### Telemedicine

Telemedicine has been the main approach to deliver pediatric health care during the COVID-19 pandemic when it is accessible, available, and appropriate to address the present problem. Only 8% of Americans had used telemedicine at one point in 2019 [19]; however, this number has significantly increased during the recent pandemic. A number of barriers to wide adoption were reported earlier, such as discomfort of patients, parents, and providers using telemedicine technology, lower reimbursement rates, and preference for in-person visits. The only exception has been people in remote or rural areas with limited access to care, especially from specialists and subspecialists [20,21]. Due to the lack of an effective vaccine or therapy for COVID-19 as well as social distancing and stay-at-home lockdown orders, exploring alternatives for in-person visits is inevitable. On March 17, 2020, the US government issued a key temporary waiver for several rules related to Health Insurance Portability and Accountability Act (HIPAA) regulations around telemedicine for both audio and video communications [22,23]. This waiver is a recognition of the value and the urgent need to use telemedicine as well as the existing high-quality evidence supporting its utility. Additionally, insurers in the United States have expanded their coverage and reimbursement of various types of home (ie, direct to consumer) telemedicine visits [22]. Telemedicine has clear benefits and unique potential for scalability for general pediatricians as well as pediatric specialists in academic, community, and private sectors as well as in urban and rural settings [24-40]. However, clinics and institutions should pay careful attention to establishing plans to provide required technical support for these services and to integrate them with their workflows. This workflow process involves not only providers but also administrative support staff, nurses, social workers, case managers, other team members, patients, and patients’ families. Telemedicine is being used more frequently in pediatric care, and a number of studies have examined its quality of care. In a home-based telehealth videoconferencing group of adolescent transplant recipients living a median of 57 miles from a transplant center, medication adherence generally improved, although technological difficulties limited participation [41]. A telemedicine approach is appropriate for several indications, ranging from prevention of long-term or chronic health conditions by promoting well-being to management of acute illness and provision of mental health services.

### Psychosocial Support

Technology itself can be used for therapeutic benefit; in children and adolescents, this application has mostly been directed toward improving mental health and decreasing substance use. A recent systematic review and meta-analysis that included 29 different programs found a medium effect size for internet-based cognitive behavioral therapy (iCBT) for depression and anxiety in children and adolescents compared to waitlist control groups [42]. At least 10 evidence-based iCBT programs are available for adolescent anxiety [43]. Importantly, these programs showed moderate to high use, especially with adjunct support from coaches, teachers, or therapists, to demonstrate credibility and to help users complete their target behavior [43]. Interestingly, even without support, when evidence-based iCBT is open-access and self-directed, engagement is lower than in controlled trials; however, many adolescents still achieved significant reductions in anxiety levels [44]. iCBT is also being adapted for more populations, such as adolescents with intellectual disability and anxiety [45]; youth insomnia [46]; sickle cell disease [47]; and chronic pain [48], including functional abdominal pain or dyspepsia [49]. The latter, in particular, was found to result in cost savings and lower health care utilization [50].

A systematic review examining the use of remotely delivered psychological therapies for chronic pain conditions in children and adolescents included 10 eligible studies [51]. These studies involved patients with headache, juvenile idiopathic arthritis, sickle cell disease, irritable bowel syndrome, and mixed pain syndromes [51]. Reduced headache severity was the only positive finding [51]. An innovative motivational intervention for young people with schizophrenia involving a virtual community of peers with schizophrenia and motivational coaches enhanced social motivation and decreased depressive symptoms [52]. Further, some interventions have been tailored with the goal of delivery at more pertinent moments; these are called just-in-time adaptive interventions (JITAIs). For example, a mobile health intervention for homeless adolescent mothers involves a wearable wristband that measures electrodermal activity as a marker of stress and times the notification of stress signals to prompt the adolescent to use emotion regulation support [53]. It also important to note that evaluating minimal clinically important, relevant, and meaningful differences or effects as a result of these psychosocial interventions, rather than only focusing on effect sizes, is a key consideration when evaluating their effectiveness [54]. Despite the overall promising outcomes related to digital psychosocial interventions, broad and public dissemination and implementation of these interventions is still limited, and more research is needed in this area.

### Supporting Preventive Behaviors

One specific population that is amenable to digital health interventions is adolescents, who are avid users of technology. Adolescence is a life period in which the majority of individuals are relatively healthy. It is also a time to establish healthy behaviors and prevent the development of future chronic illnesses. A review of less intensive SMS text messaging interventions for prevention, treatment, and knowledge outcomes for sexually transmitted infections (STIs) has shown equivocal results [55]. Additionally, other digital media interventions only showed effects on STI knowledge [56] as well as condom attitudes and self-efficacy [57]. Further, electronic STI testing was also found to be feasible and acceptable to young people and to increase uptake of testing [58]. Multiple technology interventions have been developed to address other behaviors in adolescents, such as alcohol use, smoking, and drug use. A meta-analysis of SMS text messaging interventions to reduce young adult binge drinking did not show effectiveness [59], while brief interventions and mobile applications showed some improvement in knowledge [60,61]. Intervening earlier in adolescence may provide a greater window of opportunity to enact effective, long-lasting behavior changes.

### Medication Adherence and Self-Management

Digital interventions targeting adherence and disease management provide opportunities to enhance communication between patients and their health care teams. Multiple technologies have been used to support disease monitoring and self-management outside clinical settings and outside the purview of parents. In two recent systematic reviews, adherence-promoting interventions have been shown to be efficacious among children and adolescents with or without chronic medical conditions. However, most of the studies were low to moderate in quality; most were pilot studies, only a few were randomized controlled trials, and all had variable follow-up periods [8,10]. Monitoring patients’ moods using technology may have several benefits. It can improve self-awareness of emotions and behaviors, decrease the amount of time to seek mental health help due to symptom awareness, and enhance clinicians’ understanding of their patients’ symptoms and functioning [62]. Several interventions use SMS text message–based support, such as for HIV medication adherence [63,64]; however, these interventions are not always effective [65]. Further, remote monitoring of asthma through an electronic health intervention led to similar asthma control to a usual care group, with fewer in-person visits [66]. Self-management interventions for type 1 diabetes may be more effective with clinician support [67] or when they involve videogames [68]. Further, to address childhood obesity, self-monitoring is often a key treatment component in behavioral interventions for weight loss. However, for these mobile interventions, a small but significant effect size has been reported [69]. Moreover, other novel digital interventions are being developed and tested in various health care fields, such as interactive power toothbrushes to improve plaque removal [70] and eye-gaze control technology for cerebral palsy [71].

### Peer Support

Digital approaches to optimize peer support are another interesting area of research that could improve pediatric health and well-being and also provide career and educational support. For example, for youth with physical disabilities, various formats of electronic mentoring from near peers were found to be helpful in making career decisions, coping with daily life, and advancing social skills; these benefits were a result of garnering support from other young people who understood their health challenges [72,73]. Social media has been used for healthy nutrition interventions [74], most notably for increasing desirable food consumption (fruits and vegetables) [75]; however, the effectiveness of these interventions remains unclear, and there are concerns related to social undesirability of posting health-related weight goals publicly as opposed to in private online groups [76].

## A Call for Further Research

Although the field of digital intervention continues to grow at a fast pace, several unanswered questions and knowledge gaps remain and should be explored further [77-81]. In particular, telemedicine and other digital approaches for pediatric health care delivery across different settings (eg, academic and community) are expected to continue, especially after their wide adoption during the COVID-19 pandemic. Patients, families, and providers are more comfortable with these approaches [82,83], and several financial barriers related to system adoption and reimbursement concerns have been resolved. Furthermore, in 2008, the American Academy of Pediatrics (AAP) established a Section on Telehealth Care (SOTC) [84]. In 2019, the AAP-SOTC launched a telemedicine initiative: SPROUT (Supporting Pediatric Research on Outcomes and Utilization of Telehealth) [85]. SPROUT is a Collaborative Telehealth Research Network funded by the National Institutes of Health (NIH) [85]. These efforts and the current widespread use of telemedicine in pediatrics reflect the importance and the need for further research to leverage and optimize remote and digital pediatric health care delivery.

Future studies should consider evaluating the following research areas related to telemedicine and other digital approaches: cost-effectiveness return on investment; impact on quality of care; balance of the use and number of visits needed for the management of both acute illness and chronic health conditions; system readiness for further adoption in other settings, such as inpatient services, subspecialist consultations, and rural areas; ongoing user-centered evaluations with feedback from patients, families, and health care providers; strategies to optimize health equity and address disparities in access to care related to race and ethnicity, socioeconomic status, immigration status, and for rural communities; privacy and security concerns for protected health information with HIPAA-secured programs; confidentiality issues for some specific populations, especially adolescents and people in need of mental health services; early detection of violence exposure and child neglect; and finally, integration of training into undergraduate and graduate medical education and subspecialty fellowships. Addressing these research areas is essential to understanding the benefits, sustainability, safety, and optimization strategies of telemedicine and other digital approaches as key parts of modern health care delivery. These efforts will inform long-term adoption of these approaches with expanded dissemination and implementation efforts.

## Conclusions

The aforementioned interventions only offer a glimpse into the future of technology for pediatric health; now, a pathway to their wider utilization has been established due to greatly increased need. The COVID-19 pandemic has rapidly transformed health care systems worldwide, with significant variations and innovations in adaptation. There has been rapid expansion of the leveraging and optimization of digital approaches to health care delivery, particularly integrated telemedicine and virtual health. The fight against the COVID-19 pandemic is ongoing and will continue to be a top priority for national and international health organizations as well as health care systems worldwide. Digital approaches including but not limited to telemedicine, such as those described in this viewpoint, have played and will play major roles as invaluable and reliable resources to overcome restrictions and challenges imposed during the COVID-19 pandemic and to increase access to effective, accessible, and consumer-friendly care to more patients and families. Increasing numbers of providers, nurses, administrative staff, and institutions are building experience and comfort using these digital approaches, which have undoubtedly changed the way we practice medicine. However, a number of challenges remain when optimizing and integrating digital approaches for health care delivery into the public health response to the current COVID-19 outbreak and other possible future pandemics. Regulatory changes are essential to support the safe and wide adoption of these approaches. Involving all relevant stakeholders in addressing ongoing and future challenges as well as logistical, technological, and financial barriers will be key for success. This includes support for research funding to develop a sound evidence base for the efficacy of pediatric digital interventions as well as to understand their reach to heterogeneous pediatric patient populations to limit exacerbation of health care disparities. Digital approaches to health care delivery, particularly telemedicine, are ideal strategies to optimize general pediatric and subspecialty care for all children and adolescents regardless of their location.

## Appendix

Multimedia Appendix 1 JMIR Pediatrics and Parenting (JPP) COVID-19 Call for Papers.

## Abbreviations

AAP: American Academy of Pediatrics
COVID-19: coronavirus disease
HIPAA: Health Insurance Portability and Accountability Act
iCBT: internet-based cognitive behavioral therapy
JITAI: just-in-time adaptive intervention
NIH: National Institutes of Health
SARS-CoV-2: severe acute respiratory syndrome coronavirus 2
SOTC: Section on Telehealth Care
SPROUT: Supporting Pediatric Research on Outcomes and Utilization of Telehealth
STI: sexually transmitted infection
