# Supplementary material for: Digital Approaches to Remote Pediatric Health Care Delivery During the COVID-19 Pandemic: Existing Evidence and a Call for Further Research
Source: JMIR Pediatr Parent. 2020 Jun 25;3(1):e20049. doi: 10.2196/20049 (PMC7318926; doi:10.2196/20049)
Supplement: Multimedia Appendix 1 [file pediatrics_v3i1e20049_app1.pdf]

JMIR Pediatrics and Parenting

Back to Announcements Index

# Theme Issue 2020: Digital Approaches for Pediatric Healthcare Delivery during the Coronavirus (Covid-19) Pandemic

### Call for Papers: “Theme Issue 2020: Digital Approaches for Pediatric Healthcare Delivery

### during the Coronavirus (Covid-19) Pandemic” in the JMIR Pediatrics and Parenting (JPP)

### Special Theme Issue/E-Collection

Given the current outbreak of the novel coronavirus worldwide, we have created a call for papers “Theme Issue 2020: Digital Approaches for Remote Pediatric Healthcare Delivery during the Coronavirus (Covid-19) Pandemic”

Researchers are encouraged to use the JPP Theme Issue to share interim and final research data related to the digital approaches for remote pediatric healthcare delivery in different settings, such as telemedicine, web-based interventions, mobile apps, wearable devices and other novel digital strategies.

We will reduce or waive fee if no grant funding is available.

Our goal is to rapidly and openly inform efforts aimed at optimizing care delivery for children and adolescents, with or without chronic health conditions.

All articles submitted to this theme issue will be shared and published rapidly through the following mechanisms:

JMIR Preprints (example) are immediately available after submission (with DOI); authors should select the preprint option on submission. Preprints already submitted to MedRxiv can be transmitted to JPP via the M2J interface

Free fast-tracking, rapid peer-review, and publication within 1-3 weeks

Free “PubMed Now!” feature, ensuring that the paper is submitted to and searchable on PubMed within 24 hours after acceptance (fees are waived)

Accelerated publication where we do not wait for APC payments, rather production will begin immediately after acceptance

All peer-reviewed research publications in this theme issue are immediately and permanently made open access—this is of course the standard for JMIR journals

Special tagging of the XML of the published paper for priority release on PubMed Central (in collaboration with NCBI)

Questions regarding this Theme Issue should be directed to the JPP Editor-in-Chief, Sherif Badawy, MD, MS, sbadawy@luriechildrens.org, JPP Section Editor, Ana Radovic, MD, MSc,

ana.radovic@chp.edu or the editorial team at ed-support@jmir.org.

**How to submit**

Please submit JMIR Pediatrics and Parenting by selecting "Theme Issue 2020: Digital Approaches for Remote Pediatric Healthcare Delivery during the Coronavirus (Covid-19) Pandemic" in the "Section" drop-down list.

See also "How do I submit to a theme issue?" in our knowledge base.
